# Analysis of proteomic profiles and functional properties of human peripheral blood myeloid dendritic cells, monocyte-derived dendritic cells and the dendritic cell-like KG-1 cells reveals distinct characteristics

**DOI:** 10.1186/gb-2007-8-3-r30

**Published:** 2007-03-01

**Authors:** Claire Horlock, Farouk Shakib, Jafar Mahdavi, Nick S Jones, Herb F Sewell, Amir M Ghaemmaghami

**Affiliations:** 1 Institute of Infection, Immunity and Inflammation, School of Molecular Medical Sciences, The University of Nottingham, Nottingham NG7 2UH, UK; 2 Division of Otorhinolaryngology, School of Medical and Surgical Sciences, The University of Nottingham, Nottingham NG7 2UH, UK

## Abstract

**Background:**

Dendritic cells (DCs) are specialized antigen presenting cells that play a pivotal role in bridging innate and adaptive immune responses. Given the scarcity of peripheral blood myeloid dendritic cells (mDCs) investigators have used different model systems for studying DC biology. Monocyte-derived dendritic cells (moDCs) and KG-1 cells are routinely used as mDC models, but a thorough comparison of these cells has not yet been carried out, particularly in relation to their proteomes. We therefore sought to run a comparative study of the proteomes and functional properties of these cells.

**Results:**

Despite general similarities between mDCs and the model systems, moDCs and KG-1 cells, our findings identified some significant differences in the proteomes of these cells, and the findings were confirmed by ELISA detection of a selection of proteins. This was particularly noticeable with proteins involved in cell growth and maintenance (for example, fibrinogen γ chain (FGG) and ubiquinol cytochrome c) and cell-cell interaction and integrity (for example, fascin and actin). We then examined the surface phenotype, cytokine profile, endocytic and T-cell-activation ability of these cells in support of the proteomic data, and obtained confirmatory evidence for differences in the maturation status and functional attributes between mDCs and the two DC models.

**Conclusion:**

We have identified important proteomic and functional differences between mDCs and two DC model systems. These differences could have major functional implications, particularly in relation to DC-T cell interactions, the so-called immunological synapse, and, therefore, need to be considered when interpreting data obtained from model DC systems.

## Abstract 2

Important proteomic and functional differences between peripheral blood myeloid dendritic cells, monocyte-derived dendritic cells (moDC) and KG-1 cells have been identified.

## Background

Dendritic cells (DCs) are highly specialized antigen presenting cells that originate from bone marrow progenitor cells. They represent a major cellular component of the innate immune system and their interaction with cells of the adaptive immune system (for example, T cells) is critical for initiating immune responses and maintaining tolerance [1]. DCs exist in two stages of maturation. Immature cells are found throughout the body where they act as sentinels, continuously taking up antigen and undergoing activation [2]. Activation leads to the secretion of pro-inflammatory cytokines, resulting in up-regulation of co-stimulatory molecules and migration to the lymph nodes. During their maturation, DCs lose their antigen-capturing capacity and become mature immuno-stimulatory cells that have the ability to activate naïve T cells.

There are two main DC types in human peripheral blood, known as myeloid DCs (mDCs) and plasmacytoid DCs (pDCs). mDCs are the major subset, representing around 80% of blood DCs [3]. For *ex vivo *studies, mDCs can be isolated from peripheral blood using immunomagnetic cell separation [3]. However, the main obstacle here is that DCs represent only around 1% to 3% of peripheral blood mononuclear cells (PBMCs). This has, therefore, prompted researchers to use other model systems for studying mDC biology. For instance, DCs can be generated *in vitro *from peripheral blood monocytes by culturing them for six days in the presence of interleukin (IL)-4 and granulocyte-macrophage colony stimulating factor (GM-CSF). Under such culture conditions, cells acquire an immature DC morphology and express DC differentiation antigens [4]. These monocyte-derived DCs (moDCs) are routinely used as an mDC model in DC research.

Several human monocytic cell lines are also available, including U937, THP-1, MUTZ-3, HL-60, KG1 and MM6, and some of these have been shown to be able to differentiate into DC-like cells [5-9]. KG1 cells, which acquire a DC-like phenotype after stimulation with phorbol 12-myristate 13-acetate (PMA) and ionomycin [6], are probably the most widely used in DC research. PMA- and ionomycin-stimulated KG1 cells show typical DC morphology and become adherent with long neurite processes. They also show up-regulation of major histocompatibility (MHC) class I and II molecules, co-stimulatory molecules and DC-specific markers [6]. Furthermore, they are able to stimulate allogeneic T cell proliferation at levels similar to PBMC-derived DCs [6]. It has also been shown that KG1 cells are able to cross-present exogenous antigen to CD8+ T cells and display similar regulation of MHC class II trafficking to DCs [8]. Therefore, KG1 cells are considered to be a good model system to study human DC biology.

Despite the extensive use of both moDC and KG1 cells as mDC models, their similarity to peripheral blood DCs is yet to be properly defined. This study aims to assess the suitability of both moDC and KG1 cells as model cells for peripheral blood DCs by comparing their proteomes in relation to their surface phenotypes, cytokine profiles and T cell activation ability.

## Results and discussion

**Figure 1:**
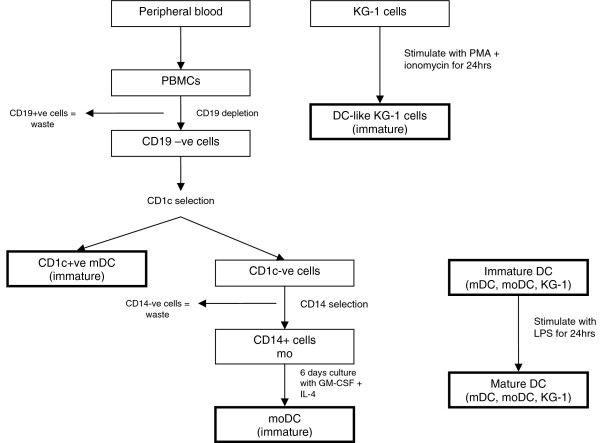
Cell culture work-flow. Overview of the methods used for isolation/generation of mDCs, moDCs and DC-like KG-1 cells.

DCs are sentinels of the immune system and play a pivotal role in bridging innate immunity with the adaptive immune response. Given the scarcity of peripheral blood DCs and the ethical and technical difficulties involved in obtaining tissue-derived DCs from human sources, investigators have resorted to using different model systems for studying DC biology. Although moDC and KG-1 cells are routinely used as mDC models [8,10,11], a thorough comparison of these cells has not yet been carried out. A number of phenotypic and functional comparisons have previously been made between mDCs and moDCs [12] and moDCs and KG-1 cells [13], but no studies have compared the proteomes of all three cell types. In this study we compare the proteomes of mDCs, moDCs and KG-1 cells, and then attempt to relate this to the functional properties of these cells. Figure 1 shows the workflow and the way in which each cell population was generated or separated.

### Dendritic cell proteomes

Proteomic data are scarce in relation to DC biology, and where available they only focus on moDCs [14-16]. Others have focused on gene expression, as well as obtaining some proteomic data, in monocytes and moDCs [17-19]. The present study compared the whole cell proteome of immature mDCs, moDCs and KG-1 cells. Clearly, a major challenge in proteomic studies of DCs is obtaining enough protein for performing two-dimensional electrophoresis. This limitation was partly overcome by using a large volume of blood (approximately 120 ml) for cell separation. We also pooled whole cell lysates of DCs from seven individuals to obtain sufficient quantities of protein and to eliminate inter-individual variations. We found that peripheral blood mDCs have six- and five-fold lower protein content per cell than moDCs and KG-1 cells, respectively (data not shown). Unfortunately, the low numbers of mDCs in peripheral blood (approximately 1% of PBMCs), together with their lower protein content, meant that, despite pooling samples, we were able to run only duplicate gels for mDCs.

**Figure 2:**
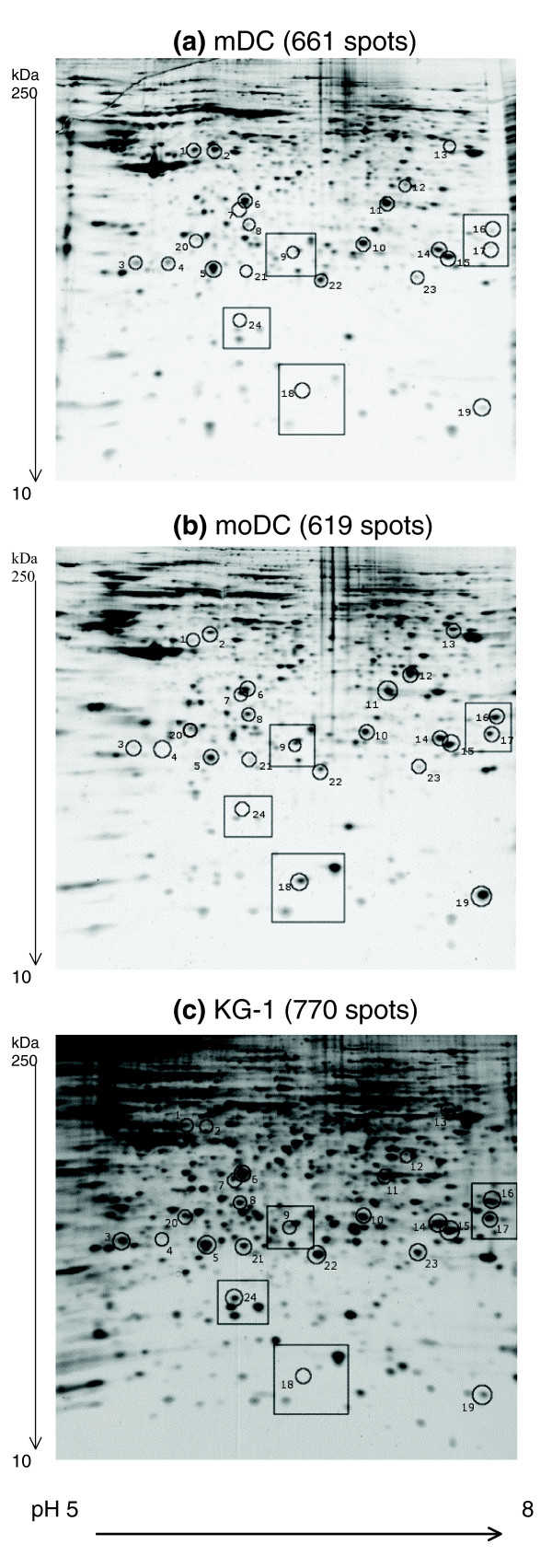
Two-dimensional electrophoresis gels. Three representative two-dimensional gel images of **(a) **mDCs, **(b) **moDCs and **(c) **KG1 cells. Whole cell lysate protein (30 μg) was applied to immobilized pH gradient strips (pH 5-8), subjected to isoelectric focusing and separated on 10% to 20% polyacrylamide gel before silver staining. Images were analyzed using PDQuest and normalized by total quantity in valid spots. Highlighted spots were excised and protein identifications attempted using MALDI-TOF mass spectrometry. Boxed areas are shown in detail in Figure 3. Further gel information and protein identifications are shown in Table 2. The experiment was repeated three times (two times in the case of mDCs) with similar results.

**Figure 3:**
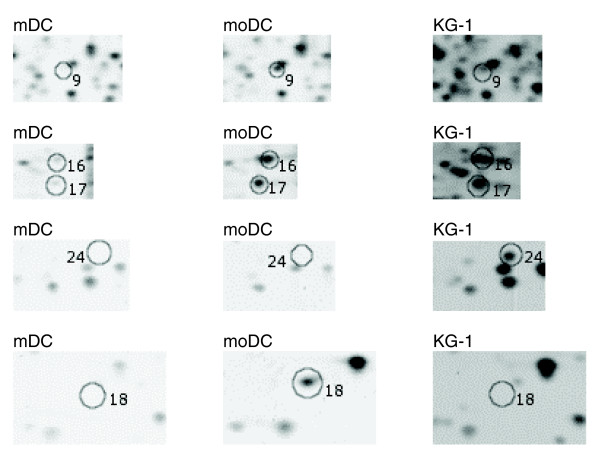
Detailed view of two-dimensional gels. Detailed areas of the mDCs, moDCs and KG-1 gels. The areas correspond to boxed areas in Figure 2.

**Figure 4:**
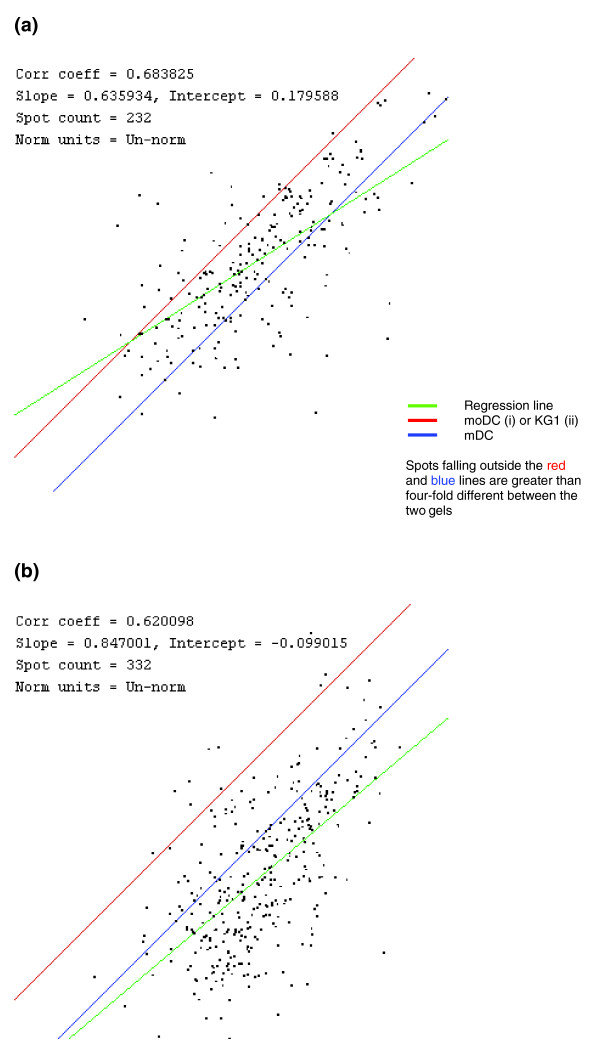
Comparison of matched spots in all three cell types. A comparison of mDCs with **(a) **moDCs and **(b) **KG-1 cells is shown by plotting the quantity of each spot in one gel (x axis) with the quantity of each spot in the second gel (y axis). The regression line generated from the plot is shown in green, and spots that fall between the red and blue lines are within two-fold higher or lower in either of the gels. A correlation coefficient was obtained from the regression line.

**Figure 5:**
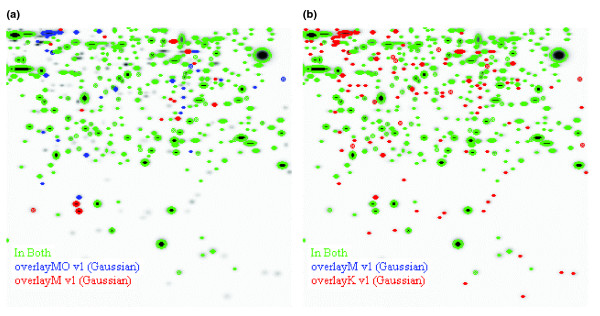
Overlaid gel images. Gaussian images of mDCs were overlaid with those of either **(a) **moDCs or **(b) **KG-1 cells. This reveals differences in the proteomes, with some unique spots.

Figures 2 and 3 show three representative two-dimensional gel images of the different cell types. Gel images were analyzed using PDQuest software and all images were normalized before any comparisons between gels were made. The total number of spots in the gels were 661, 619, and 770 for mDCs, moDCs and KG-1 cells, respectively. To analyze the comparability of gels, the densities of spots matched in all three gels were plotted and a correlation coefficient value was calculated. The proteome of mDCs showed different levels of similarity compared with those of moDCs and KG-1 cells (correlation coefficient 0.68 and 0.62, respectively) (Figure 4). Duplicate gels of mDCs were reproducible (correlation coefficient >0.90), as were triplicate gels of moDC and KG-1 cells. Figure 5 shows an overlay of Gaussian images of mDCs, moDCs and KG-1 cells.

Thirty-five spots were up-regulated more than four-fold in mDCs compared with the DC models, and fifty were down-regulated by the same amount (Table 1). A number of differentially expressed proteins, which appeared to be more than two-fold different in intensity (either up- or down-regulated) in the DC models compared to mDCs, were excised from the gels and subjected to trypsin digestion and MALDI-TOF (matrix-assisted laser desorption/ionisation-time of flight) mass spectrometric analysis; Table 2 shows the corresponding protein data. The factor of difference was calculated by dividing the intensity of the protein spot in mDCs by that of the corresponding spot in moDCs or KG-1 cells. Eighteen spots were successfully identified using MALDI-TOF mass spectrometry. These proteins are known to be involved in a wide spectrum of biological processes, including functions related to cell integrity and metabolism (Table 2).

**Table 1:** Summary of gel spot data

Cell type	Total spots	Matched spots	No. of spots >4-fold higher in mDCs	No. of spots <4-fold lower in mDCs
mDC	661	661	NA	NA
moDC	619	550	35	50
KG-1	770	562	60	114

NA, not applicable.

**Table 2:** Identifications of differentially expressed spots in mDCs, moDCs and KG-1 cells using MALDI-TOF mass spectrometry

Spot no.	Protein identification	Accession no. (Swiss-Prot)	Induction factor* (mDC/moDC)	Induction factor* (mDC/KG-1)	Theoretical pI/Mr	Sequence coverage (%)	MASCOT score	Biological process^†^
1	Fibrinogen γ chain	P02679	8.60	3.28	5.37/51,511.66	17	91	1,8,12,13
2	Ubiquinol-cytochrome-c-reductase complex core protein 1	P31930	24.41	21.01	5.94/52,618.79	13	58	1,2
3	-	-	8.05	-2.27	-			
4	-	-	Not in mo	21.06	-			
5	Glutathione S-transferase P	P09211	2.49	1.42	5.44/23,224.64	40	102	1, 2, 9
6	L-lactate dehydrogenase B chain	P07195	1.70	-1.28	5.72/36,507.30	33	175	2
7	-	-	Not in m	Not in m	-			
8	-	-	-4.06	9.51	-			
9	Pyridoxal kinase	O00764	Not in m	Not in m	5.75/35,102.30	22	96	2
10	Nuclease isoform Sm3	P13717	1.54	2.01	6.95/28,944.65	56	191	1, 2
11	Annexin A1	P04083	1.43	2.26	6.64/38,583.05	59	202	1, 4, 5, 7, 9
12	Fructose-1,6-bisphosphatase	P09467	-14.34	2.62	6.54/36,818.12	48	205	1, 2
13	Fascin	Q16658	-8.78	-7.87	6.81/54,398.81	23	133	4,5,6
14	Phosphoglycerate mutase I	P18669	-1.37	-2.21	6.75/28,672.74	47	143	1, 2
15	Triosephosphate isomerase	P60174	1.02	-1.14	6.51/26,538.30	56	209	2
16	Actin related protein 2/3 complex subunit	O15144	-16.74	-20.03	6.84/34,333.02	19	91	4,5,6, 9
17	Carbonic anhydrase II	P00918	-15.63	-10.99	6.86/29,114.86	29	88	1, 2
18	Phosphoglucomutase I	Q16106	-33.05	-2.51	5.33/11,328.66	28	40	2
19	Cystatin B	P04080	-30.68	-2.13	6.96/11,139.59	34	61	1, 2, 9
20	-	-	-7.27	-3.69	-			
21	-	-	-3.03	-22.70	-			
22	Chain A, crystal structure of human Dj-1	Q99497	1.85	-2.02	6.33/19,891.05	36	89	1, 7
23	Proteasome beta 2 subunit	Q9BWZ9	-1.07	-5.82	6.52/22,840.06	26	77	1,2,9
24	dUTP pyrophosphatase isoform 2	P33316	Not in m or mo	Not in m or mo	9.65/26,706.39	35	93	1,2, 9

MASCOT scores >64 were taken to be significant (*p *< 0.05). *Induction factor corresponds to the factor of difference between spot volume in mDCs compared with the respective mDC model. ^†^Biological process: 1, cell growth/maintenance; 2, metabolism; 3, cell communication; 4, morphogenesis; 5, response to external stimulus; 6, cell motility; 7, response to stress; 8, circulation; 9, regulation of cellular processes; 10, cell differentiation; 11, death; 12, cell death; 13, coagulation; 14, homeostasis.

The majority of the proteins that showed higher levels of expression in mDCs are known to be involved in cell growth and maintenance, including FGG, ubiquinol cytochrome c reductase, glutathione S transferase, nuclease isoform sm3 and annexin A1. Some of these differentially expressed proteins also appear to be involved in DC maturation. Pereira *et al*. [15] have shown higher expression of FGG in the proteome of immature moDCs compared to mature moDCs. Furthermore, fascin and actin, which showed substantially lower expression (8- and 16-fold, respectively) in mDCs compared with both DC models, are known to play important roles in maintaining cell structure and in the formation of immunological synapses between DCs and T cells [20-22]. Al-Alwan *et al*. [21] have previously shown that increased fascin expression correlates with DC maturation state, and recent work supports this, suggesting that fascin is a mature DC marker [23]. This, together with our data on FGG expression, suggests that, at least in their resting state, mDCs have a less mature phenotype compared to moDCs and KG-1 cells.

### Dendritic cell lysate ELISA

**Figure 6:**
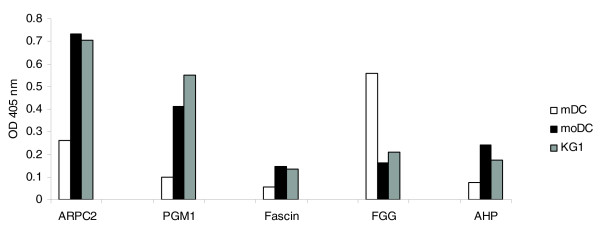
ELISA detection of cell lysate proteins. Differentially expressed digoxigenin-labeled proteins of mDCs, moDCs and KG-1 cell lysates were captured by specific antibody coated plates and detected with a polyclonal anti-dioxigenin Fab fragment. Data are representative of two experiments.

To confirm the proteomics data, we used a capture ELISA to assess the relative expression of five proteins that had an induction factor greater than two, namely actin related protein 2/3 complex 2 (ARPC2), phosphoglucomutase 1 (PGM1), fascin, FGG and carbonic anhydrase 2 (CAH2) (Table 2). The pattern obtained was in general agreement with the proteomes obtained for each of the three cell types. Thus, as expected, ARPC2, PGM1, fascin and CAH2 were found to be lower in mDCs compared to the two models, whereas FGG was higher (Figure 6).

### Cell surface marker expression

We compared the three cell types by studying their surface phenotypes. Immature cells were cultured in the presence of lipopolysaccharide (LPS) for 24 h to produce a mature cell type. The cell markers used for characterization were CD11c, CD40, CD62L, CD80, CD83, CD86, CD206, CD209, HLA DR and Toll-like receptor (TLR)-4, which have all been reported to be found on dendritic cells [4,24].

**Figure 7:**
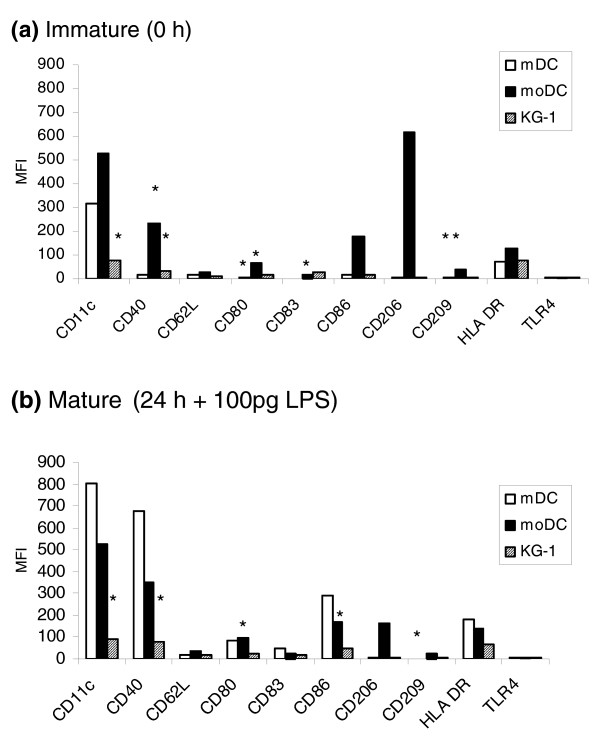
Phenotypic comparison of cells. The expression of cell surface markers on **(a) **immature and **(b) **mature mDCs, moDCs and KG1 cells. Immature cells were freshly isolated mDCs, moDCs, on day 6 of culture, and KG-1 cells stimulated with PMA and ionomycin for 24 h. Cells were matured in the presence of LPS (100 pg/ml) for 24 h. Shown are the mean fluorescence intensities of four individual experiments. Background levels of staining were determined using isotype controls. A Student *t*-test was carried out to determine the significance of the data (*p < 0.05).

As with our proteomic data, cell surface marker expression suggested that immature mDCs expressed lower levels of the usual DC maturation markers compared with both moDCs and KG-1 cells. The mDC models, moDcs and KG-1 cells, expressed significantly higher levels of CD11c, CD40, CD80, CD83 and CD209 than mDCs (Figure 7). However, mDCs showed significant up-regulation of the classic DC maturation markers CD40, CD80, CD83 and CD86 after 24 h stimulation with LPS; levels of these markers were more than ten-fold higher in mature compared to immature cells. The mDC models also showed up-regulation of these markers, but to a lesser extent (more than three-fold). The expression of cell surface markers on mature KG-1 cells was lower than on both mDCs and moDCs, with CD11c, CD40, CD80 and CD86 being expressed at significantly (*p *< 0.05) lower levels than on mDCs (Figure 7).

Myeloid DCs showed a more mature phenotype after stimulation with LPS (as shown by higher expression of CD40, CD80, CD83 and CD86) compared with moDCs and KG-1 cells. Interestingly, the mannose receptor (CD206), which has important functions in endocytosis, antigen recognition and binding and MHC class II presentation [25-27], was only detectable on moDCs and was down-regulated by 4-fold after stimulation with LPS for 24 h; only negligible levels were found on mDCs and KG-1 cells. This would, therefore, suggest that in *in vitro *assays, moDCs could bind and internalize certain antigens, particularly glycoproteins, more efficiently. These findings are in keeping with those of Hajas *et al*. [13] showing that moDCs express much higher levels of CD206 than KG-1 cells and they could internalize antigens relatively more efficiently. The expression of DC-SIGN (DC-specific intercellular adhesion molecule-3-grabbing non-integrin or CD209) was low on all three cell types, but significantly higher on immature moDCs and KG-1 cells compared with mDCs.

Our finding of negligible levels of TLR4 on all three cell types is somewhat different from those of others [28,29] who found no expression of TLR4 on mDCs, but did show expression on moDCs. However, there are studies showing TLR4 expression by both mDCs and moDCs, but not on pDCs [30]. This discrepancy in data could have been caused by the use of different monoclonal antibodies and experimental conditions.

### Cytokine expression profile

**Figure 8:**
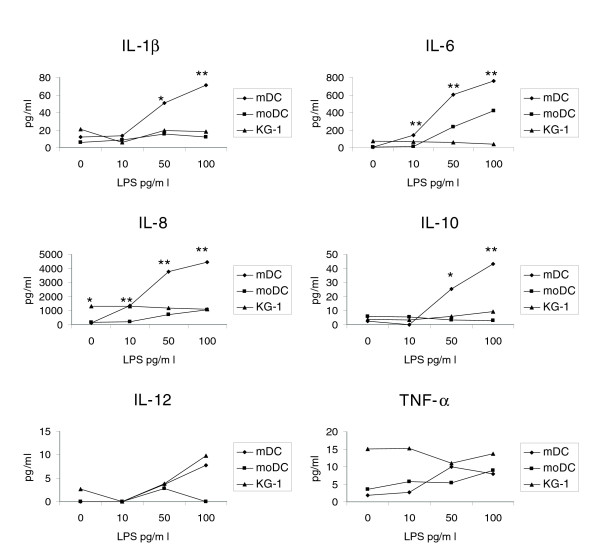
Cytokine expression profiles of cells. Cytokine production by mDCs and the DC models in response to LPS. Cytokine concentrations were measured by CBA and analyzed using the cytometric CBA analysis software; with further analysis in Excel. Data shown are the means of triplicate experiments. A Student *t*-test was carried out to determine the significance of the data (*p < 0.05, **p < 0.01).

Peripheral blood mDCs were found to express significantly higher levels of key inflammatory (IL-1β, IL-6 and IL-8) and regulatory (IL-10) cytokines, compared to moDCs and KG-1 cells. Levels of IL-1β, IL-6, IL-8 and IL-10 were dose dependent, and following 24 h culture with either 50 or 100 pg/ml LPS were significantly higher in mDCs than in moDC and KG-1 cells (Figure 8). The IL-6, IL-10 and IL-12 data are at variance with a previous study [12], but this may be due to the use of different stimuli (for example, intact *Escherichia coli *rather than LPS), culture conditions and cytokine detection method by the authors. This pattern of cytokine production clearly makes mature mDCs more efficient in the cross-talk with T cells [31,32] and other cells of the innate immune system (for example, natural killer cells), as well as in exerting inflammatory and/or regulatory effects mediated through cytokine production. This again is in line with our proteomic data suggesting that mDCs have a less mature phenotype, at least in their resting state, compared to the two DC models, moDCs and KG-1 cells [33].

### Functional analysis

**Figure 9:**
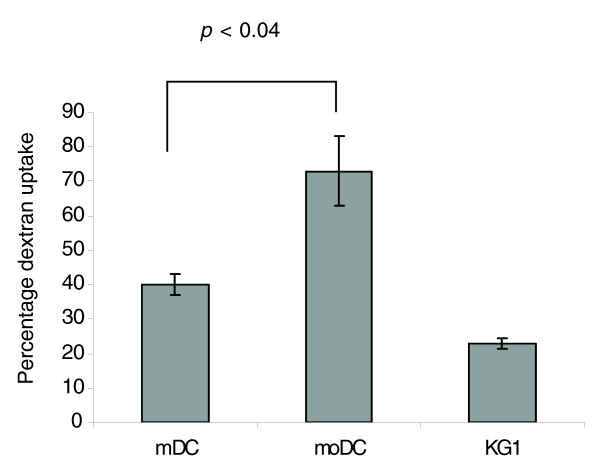
Analysis of endocytic activity using a FITC-dextran uptake assay. Freshly isolated mDCs, moDCs and KG-1 cells were pulsed with dextran for 1 h at 37°C and the uptake was measured by flow cytometry. Data represent average values of three experiments for each cell type.

**Figure 10:**
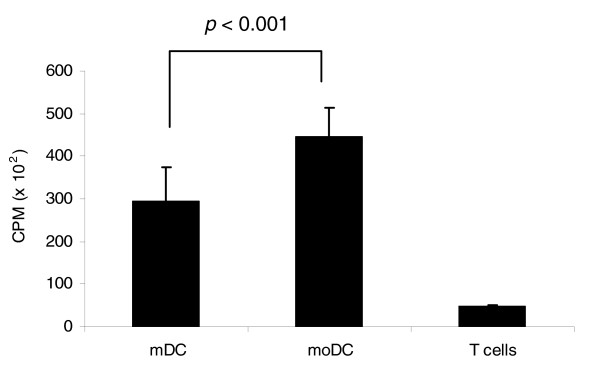
T cell activation assay. Autologous T cells were cultured in the presence of mDCs, moDCs or KG-1 cells in a 1:10 DC:T cell ratio for 3 days, followed by an 18 h pulse with [^3^H]thymidine. Thymidine incorporation was measured in a scintillation counter. Data represent the average of quadruplicate experiments.

Having demonstrated that unstimulated moDCs have a more mature phenotype than freshly isolated mDCs, as shown by proteomics (for example, lower FGG and higher fascin) and surface marker expression (higher CD83), we then proceeded to assess the endocytic and T cell stimulatory abilities of the DCs using dextran uptake and autologous mixed leukocyte reaction, respectively. moDCs were found to be better in endocytosis (Figure 9) and T cell activation (Figure 10) compared to mDCs, and this is in keeping with their more advanced maturation status. Others have shown [12] that, upon stimulation, both mDCs and moDCs are equally efficient in autologous T cell activation, which is in agreement with our finding that mDCs acquire a fully mature phenotype after LPS stimulation (Figure 7).

## Conclusion

Despite the general similarities between mDCs and the two DC model systems, moDCs and KG-1 cells, our findings identified important differences between the proteomes of these cells, and the findings were confirmed by ELISA detection of a selection of proteins. These differences were particularly noticeable with proteins involved in cell growth and maintenance, as well as those involved in cell-cell interaction, cell integrity and maturation. The scarcity of peripheral DCs meant that we were not able to focus on less abundant proteins in the current study, which could identify differentially expressed proteins involved in other cell functions. The functional relevance of differentially expressed proteins was confirmed by analysis of surface marker expression, cytokine profile, endocytic and T cell activation abilities of the cells, again suggesting differences in the maturation status between mDCs and the DC models. These observations have important functional implications, particularly in relation to DC-T cell interactions, the so-called immunological synapse, and, therefore, need to be considered when interpreting data obtained from model DC systems. This study clearly shows the value of the proteomic approach as a tool for studying the biology of immune cells.

## Materials and methods

### Cell cultures and stimulation

Heparinized whole blood from healthy volunteers (obtained with prior consent and Ethical Committee approval) was used for separation of PBMCs on a Histopaque density gradient (HISTOPAQUE-1077, Sigma, Poole, UK). CD1c^+ ^peripheral DCs were isolated using the CD1c dendritic cell isolation kit from Miltenyi Biotech (Bisley, UK). Briefly, this involved depleting PBMCs of CD19^+ ^B cells followed by positive selection of CD1c^+ ^cells. CD14^+ ^monocytes were isolated by positive selection from the CD1c^- ^cell fraction, and immature CD1a^+^CD83^- ^moDCs were generated as previously described [24]. Briefly, this involved culturing CD14^+ ^monocytes in the presence of IL-4 (250 IU/ml; R&D systems, Oxford, UK) and GM-CSF (50 ng/ml; R&D systems) for six days. Cells were cultured at 1 × 10^6^/ml in RPMI 1640 medium (Sigma) supplemented with 2 mM L-glutamine, 100 U/ml penicillin, 100 U/ml streptomycin (Gibco Life Technologies, Paisley, UK) and 10% (v/v) fetal calf serum (FCS; Harlan Sera-Lab, Loughborough, UK) at 37°C in 5% CO_2_. On day 3, cultured cells were fed with fresh medium containing relevant cytokines.

The human monocytic cell line KG1 was purchased from ECACC (Salisbury, UK). Cells were maintained at 0.35 × 10^6^/ml in Iscoves modified Dulbecco's medium (Sigma) supplemented with 2 mM L-glutamine, 100 U/ml penicillin, 100 U/ml streptomycin (Gibco Life Technologies) and 10% (v/v) FCS (Harlan Sera-Lab) at 37°C in 5% CO_2_. Cells were stimulated with 10 ng/ml PMA and 100 ng/ml ionomycin (both from Sigma) for 24 h, as described previously [6].

Immature peripheral mDCs, immature moDCs and DC-like KG1 cells were cultured in 48-well culture plates at 0.25 × 10^6 ^cells/ml. Four conditions were set up in parallel, stimulating cells with 0, 10, 50 or 100 pg/ml LPS (Sigma). After 24 h, 250 μl of supernatant was collected and frozen at -80°C, and cells were harvested for cell surface marker staining.

### Proteomics

#### Two-dimensional electrophoresis

Immature DC like KG1 cells, moDCs and mDCs were harvested and resuspended in lysis buffer containing 7 M urea (Fisher Scientific, Loughborough, UK), 2 M thiourea (Sigma), 4% 3- [(3-Cholamidopropyl)dimethylammonio]-1-propanesulfonate (CHAPS) (Fisher Scientific), 50 mM dithiothreitol (DTT; Fisher Scientific), 5 mM TBP (Bio-Rad, Hercules, USA), 0.5% carrier ampholytes (Invitrogen, Paisley, UK), 1× protease inhibitor (Amersham, Little Chalfont, UK), 150 U/ml benzonase (Novagen, Merck biosciences, Nottingham, UK) and a trace of bromophenol blue (Sigma). Samples were frozen at -80°C until processing. Cell lysates from seven individuals were combined and a protein concentration assay (2D Quant Kit, Amersham) was carried out. Samples of 30 μg were made up to 320 μl with lysis buffer, vortexed for 5 minutes at room temperature and centrifuged at 14,000 rpm for 30 minutes.

Immobilised pH gradient (IPG) strips (Bio-Rad) were passively rehydrated by the protein samples at 20°C for approximately 17 h. A low voltage run at 50 V was then performed for 6 h. Isoelectric focusing was run with the following conditions: rapid ramping 250 V for 15 minutes, 10,000 V for 3 h followed by total 60,000 V/h and a subsequent 500 V hold. IPG strips were equilibrated for 30 minutes in equilibration buffer, containing 6 M urea, 2% SDS, 0.05 M Tris and 20% glycerol (Fisher Scientific) for 15 minutes with 2% DTT and 15 minutes with 2.5% iodoacetamide (Bio-Rad). The second dimension separation was carried out on precast vertical 10% to 20% SDS-polyacrylamide gels (BioRad). Gels were typically run at 20 mA per gel for 18 h. Gels were stained using the Dodeca Silver Stain Kit (Bio-Rad).

#### Gel imaging and analysis

Gels were scanned on a GS-800 calibrated imaging densitometer (Bio-Rad). Gel images were analyzed using PDQuest gel analysis software version 7.1 (Bio-Rad). Spots were automatically detected, and then visually checked for undetected or incorrectly detected spots. All images were normalized according to total quantity in valid spots in each gel before any comparisons were made.

### Mass spectrometry

#### In gel digestion

Gel pieces were excised and placed in a 96-well plate, then loaded onto a MassPrep robotic liquid handling system (Waters Corporation, Elstree, UK). This was used to destain gel pieces, reduce and alkylate cysteine residues using DTT and iodoacetamide, carry out an in-gel tryptic digest and extract the resulting peptide mixture into a 96-well PCR plate. The extracted peptide mixture was manually desalted using C18 loaded zip-tips (Millipore, Watford, UK). We routinely spotted 2 μl onto sample wells of a stainless steel MALDI target plate previously spotted with 1 μl matrix solution, comprising 1 mg/ml α-cyano-4-hydroxycinnaminic acid (Sigma) in 50% acetonitrile, 50% ethanol and an internal standard, adeno corticotophic hormone (Sigma), at a final concentration of approximately 100 fmol/μl in 0.1% formic acid (Romil, Cambridge, UK). Samples were left to air dry and the plate placed in the MALDI mass spectrometer.

#### MALDI-TOF mass spectrometry analysis

Samples were analyzed using a MALDI TOF mass spectrometer (Waters Corporation) operating at a resolution of greater than 10,000 full width at half maximum in reflectron mode. Spectra were acquired at 5 Hz using a nitrogen laser (337 nm wavelength). Typically, ten data collection events were combined to generate each spectrum. Data acquisition was achieved by randomly sampling from the target well.

#### Mass spectrometry data analysis

Peak lists were entered into MASCOT PMF [34] and Expasy [35] database search engines. Search parameters included a peptide mass accuracy tolerance of 0.2 Da and allowed for modifications such as alkylation of cysteine during the tryptic digest procedure and the possible formation of methionine sulfoxide.

### ELISA

Immature mDCs, moDCs and KG1 cells were generated as described earlier. Cells were harvested and washed three times in 1 ml of 0.05% PBS/Tween blocking buffer (300 g for 5 minutes). Cell pellets were resuspended in 500 μl carbonate buffer (pH 8.6, 7.6 mM Na_2_CO_3_, 142 mM NaHCO_3_) and sonicated for 5 minutes. The sonicated cells were labeled by incubation with 20 μg digoxigenin (Roche, Basel, Switzerland) for 1 h at room temperature. The remaining free digoxiginin was neutralized with 150 mM Tris followed by dialysis against PBS (pH 7.2) overnight. The protein concentration for each cell type was measured at 280 nm using a Nanodrop (Agilent Technologies, Berkshire, UK). In the ELISA, anti-PGM1 (Abnova, Taipei, Taiwan), anti-fascin (Santa Cruz Biotechnologies, Santa Cruz, CA, USA), anti-CAH2 (Abnova), anti-FGG (Abnova) and anti-APRC2 (Abnova) antibodies (10 μl/ml) were diluted in carbonate buffer and plated onto a Nunc Immobilizer™ Amino 96-well plate n amino-reactive 96-well (Nunc, Roskilde, Denmark). Plates were incubated for 2 h at room temperature with shaking at 200 rpm. Liquid was removed from the plates and the plates were washed three times with PBS/Tween. The plate was incubated with 100 μl of each cell suspension (10 μg/ml total protein) for 2 h at room temperature. The plate was then washed 3 times with PBS/Tween and incubated with peroxidase-conjugated polyclonal anti-dioxigenin Fab fragment (Roche) at 1:5,000 in 1% BSA in PBS/Tween at 100 μl per well. Plates were incubated at room temperature for 1 h and washed 3 times as above. ABTS^® ^Peroxidase Substrate (100 μl at 5 mg/ml) (Roche) was added to each well and 30 minutes later the absorbance was measured at 405 nm.

### Phenotype and cytokine expression

#### Cell surface marker expression

The phenotypes of mDCs, moDCs and KG1 cells were analyzed using a selection of monoclonal antibodies. Mouse antibodies to human CD11c PE (clone BU15), CD40 PE (clone MAB89), CD62L FITC (clone DREG56), CD80 FITC (clone MAB104), CD83 PC5 (clone HB15a), CD86 PE (clone HA5.2B7), CD206 PE (clone 3.29B1.10) and HLA DR PC5 (clone IMMU-357) were purchased from Coulter Immunotech (Luton, UK). Mouse anti-human CD209 PE (clone DCN46) was purchased from Becton Dickinson (Oxford, UK). Mouse anti-human TLR4 PE (clone HTA125) was purchased from Serotec (Oxford, UK).

Cells were stained following 0 h and 24 h culture. Cells were washed twice in PBS (Gibco, Invitrogen), supplemented with 2% FCS, incubated with antibody for 20 minutes at 4°C, washed twice and fixed in 0.5% formaldehyde. Samples were analyzed on an EPICS Altra flow cytometer (Beckman Coulter, Luton, UK) within six days of staining. Data were analyzed using WinMDI version 2.8. [36]. Isotype-matched irrelevant antibodies were used to verify the staining specificity.

#### Cytokine expression

Culture supernatants from 4 independent experiments were collected after 24 h stimulation with 0, 10, 50 or 100 pg/ml LPS. Supernatants for each condition were pooled and a cytokine bead array (CBA; Inflammation kit, Becton Dickinson) was performed in triplicate.

### Endocytosis assay

For the analysis of the endocytic activity of the three cell types, 1 × 10^5 ^cells were incubated with FITC-dextran (40,000 MW; Sigma) for 1 h at 37°C. As a control, 1 × 10^5 ^cells were cooled to 4°C prior to incubation with dextran at 4°C for 1 h. Cells were washed three times and immediately analyzed on a FACS EPICS Altra cytometer.

### T cell activation assay

Human PBMCs were obtained as described above. Un-touched T cells were then purified by negative selection (Pan T cell isolation kit, Miltenyi Biotech) to a purity of >95%. Cells were resuspended in RPMI 1640 medium (Sigma) supplemented with 2 mM L-glutamine, 100 U/ml penicillin, 100 U/ml streptomycin (Gibco Life Technologies) and 10% (v/v) FCS (Harlan Sera-Lab). Autologous T cells (1 × 10^5^) were cultured in the absence (medium alone) or presence of 1 × 10^4 ^irradiated (3,000 rad ^137^Cs) immature mDCs and moDCs in 96-well U-bottomed microplates (Nunc) in 200 μl of medium per well. Cells were cultured for 72 h followed by an 18 h pulse with 1 μCi (0.037 MBq) of [^3^H]thymidine (Amersham Life Science, Buckingham, UK). Cells were transferred to a Unifilter-96 plate GF/C using a cell harvester and [^3^H]thymidine incorporation was measured in scintillation fluid (Microscint O) using a sinctillation counter (Canberra Packard Limited, Pangbourne, UK). All determinations were carried out in quadruplicate.

### Flow cytometric analysis

Cell surface marker expression was analyzed using WinMDI version 2.8 [36] and 7,000 live cells were gated for each analysis. The cytokine bead array data were analyzed using the BD CBA software (Becton Dickinson).

### Statistical analysis

The paired Student *t*-test was used to compare surface marker expression and cytokine expression between mDCs, moDCs and KG-1 cells. Data with *p *values of less than 0.05 or 0.01 were taken to be significant.
